# The Superoxide Dismutase Gene Family in *Nicotiana tabacum*: Genome-Wide Identification, Characterization, Expression Profiling and Functional Analysis in Response to Heavy Metal Stress

**DOI:** 10.3389/fpls.2022.904105

**Published:** 2022-05-06

**Authors:** Chunsong Huo, Linshen He, Ting Yu, Xue Ji, Rui Li, Shunqin Zhu, Fangyuan Zhang, He Xie, Wanhong Liu

**Affiliations:** ^1^Chongqing Key Laboratory of Industrial Fermentation Microorganism, School of Chemistry and Chemical Engineering, Chongqing University of Science and Technology, Chongqing, China; ^2^School of Life Sciences, Southwest University, Chongqing, China; ^3^Tobacco Breeding and Biotechnology Research Center, Yunnan Academy of Tobacco Agricultural Sciences, Kunming, China

**Keywords:** tobacco, superoxide dismutase (SOD), heavy metal, expression profiles, functional analysis

## Abstract

Superoxide dismutases (SODs) play an important role in protecting plants against ROS toxicity induced by biotic and abiotic stress. Recent studies have shown that the SOD gene family is involved in plant growth and development; however, knowledge of the SOD gene family in tobacco is still limited. In the present study, the SOD gene family was systematically characterized in the tobacco genome. Based on the conserved motif and phylogenetic tree, 15 *NtSOD* genes were identified and classified into three subgroups, including 5 *NtCSDs*, 7 *NtFSDs* and 3 *NtMSDs*. The predicted results of the transport peptide or signal peptide were consistent with their subcellular localization. Most *NtSOD* genes showed relatively well-maintained exon-intron and motif structures in the same subgroup. An analysis of *cis*-acting elements in *SOD* gene promoters showed that *NtSOD* expression was regulated by plant hormones, defense and stress responses, and light. In addition, multiple transcription factors and miRNAs are predicted to be involved in the regulation of *NtSOD* gene expression. The qPCR results indicated specific spatial and temporal expression patterns of the NtSOD gene family in different tissues and developmental stages, and this gene family played an important role in protecting against heavy metal stress. The results of functional complementation tests in the yeast mutant suggested that *NtCSD1a*, *NtFSD1e* and *NtMSD1b* scavenge ROS produced by heavy metal stress. This study represents the first genome-wide analysis of the NtSOD gene family, which lays a foundation for a better understanding of the function of the NtSOD gene family and improving the tolerance of plants to heavy metal toxicity.

## Introduction

Increasingly severe heavy metal pollution has exerted serious effects on crop growth, yield and quality. However, heavy metals such as Cu, Zn, Fe and Mn are essential for plant growth and development as micronutrient elements but become toxic when present in excess concentrations. Moreover, trace amounts of non-essential elements such as Cd and Hg are highly toxic to plants. For example, exposure of plants to Cd triggers severe symptoms, including chlorosis, root tip browning, stunted growth, and even plant death (Nagajyoti et al., 2010). Generally, heavy metals cause harmful physiological processes in plant cells, including the induction of reactive oxygen species (ROS) generation by changing the intracellular antioxidant defense system, binding the sulfhydryl, histidine and carboxyl groups of proteins and inactivating proteins. The substitution of essential ions at specific sites of proteins causes a loss of function (Hossain et al., 2012). Oxidative stress induced by heavy metals causes oxidative damage to cell membranes, proteins and nucleic acids and even increases cell death (Mittler, 2002). Therefore, scavenging excessive ROS in plant cells is an important strategy for plants to resist the toxicity induced by heavy metals.

Maintaining an optimal intracellular ROS level is essential for plant growth and development (Mittler, 2017). Generally, the ROS generated in chloroplasts, mitochondria and other organelles are maintained in a stable balance by the antioxidant defense system (ADS) in plant cells. The ADS in plants usually contains two categories of molecules: non-enzymatic antioxidant active substances, including ascorbic acid, glutathione, phenolic acid, and flavonoids; and antioxidants, such as superoxide dismutase and catalase. The excessive accumulation of intracellular ROS is scavenged by the complex ADS in plant cells. Among antioxidant systems, superoxide dismutase (SOD, EC 1.15.1.1) plays a pioneer role in scavenging ROS by activating a series of biochemical processes. Its main biofunction is to convert superoxide radicals into oxygen and hydrogen peroxide and protect plant cells from oxidative damage (Su et al., 2021). SODs are metalloproteinases, which are usually encoded by a gene family. According to the different metal cofactors, the SOD family genes in higher plants are classified into three subfamilies: copper zinc SOD (CSD), ferrum SOD (FSD) and manganese SOD (MSD) (Fink and Scandalios, 2002; Abreu and Cabelli, 2010). CSD is most widely distributed in the cytoplasm, chloroplast, peroxisomes, glyoxalic acid cycle and extracellular space. FSD is mainly located in chloroplasts, while MSD is usually located in the mitochondrial matrix and peroxisome (Pilon et al., 2011). In addition to scavenging ROS, SODs also play important roles in electron transport, photosynthesis and signal transmission. For example, CSD loss-of-function in *Arabidopsis* results in significant inhibition of plant growth and development and decreased chloroplast size, chlorophyll content and photosynthetic activity compared with the wild-type plant (Rizhsky et al., 2003). The *Arabidopsis thaliana fsd1* mutant extends fewer lateral roots than the WT strain, but root growth was resumed by expressing FSD (Dvořák et al., 2020). Based on these results, SOD plays an important role in plant growth and responses to environmental changes by maintaining ROS homeostasis.

Superoxide dismutase plays a key role in plant resistance to drought, cold, salinity, and heavy metal toxicity, and regulates plant senescence (Sahu et al., 2017; Pan et al., 2019; Zang et al., 2020; Liu et al., 2021). In recent years, a positive correlation was observed between plant metal toxicity tolerance and antioxidant enzyme activity in heavy metal stress studies (Hasanuzzaman et al., 2020). For example, Ni stress significantly activated the antioxidant enzymes such as SOD and glutathione peroxidase (GPX) in rice leaves (Hasanuzzaman et al., 2019). Cd stimulates the activity of the antioxidant enzymes SOD, CAT and POD in *Pseudochlorella pringsheimii* to scavenge heavy metal-induced ROS and exhibits a dose-dependent effect (Ismaiel and Said, 2018). Over-expression of the *SaCSD* gene from *Sedum alfredii* in *Arabidopsis* increases Cd tolerance in transgenic plants by scavenging ROS (Li et al., 2017). However, the response of different SOD isozymes to heavy metal stress differed. Significantly higher activity of CSD was observed in the leaves and roots of *Tagetes patula* under Cd stress, while MSD activity was lower (Liu et al., 2011). In *Pisum sativum* plants, the activity of CSD localized in the cytoplasm was significantly inhibited by Cd, and FSD was more resistant than CSD, while MSD, the most resistant isoform to Cd, maintained 50% activity under 40 μM Cd stress (Sandalio et al., 2001). Therefore, an in-depth understanding of the fine-tuned mechanisms of ROS homeostasis depends on systematic studies of the functions of plant SOD gene family members.

Genome-wide identification of the SOD gene family has been performed in several plants, including monocots such as rice (Yadav et al., 2019), wheat (Jiang et al., 2019), and sorghum (Filiz and Tombuloğlu, 2015), and dicots such as cotton (Wang et al., 2016), alfalfa (Song et al., 2018), and rapeseed (Su et al., 2021). Tobacco is the most widely cultivated non-food cash crop worldwide. To date, studies on the genome-wide identification of the SOD gene family in tobacco have not been reported. In the present study, the SOD gene family of tobacco cultivar TN90 was identified at the whole genome level, and its sequence characteristics and gene structure were systematically analyzed. The promoter sequences and *cis*-acting elements of SOD gene family members were predicted and analyzed. Transcription factors and miRNAs that may be involved in the regulation of *SOD* gene expression were analyzed. The tissue expression pattern and induced expression pattern of the *NtSOD* genes in plants under different metal stresses were analyzed using qPCR to further explore the function of the *NtSOD* genes. This study laid a foundation for further study of *SOD* gene function in tobacco exposed to heavy metal stress.

## Materials and Methods

### Identification and Sequence Analysis of the NtSOD Gene Family

The annotation information of genomic coding and protein sequences of *N. tabacum* cultivar TN90 were downloaded from the NCBI Genome database^1^. The HMM profiles of CSD (PF00080), FSD (PF02777) and MSD (PF00081) were downloaded from the Pfam database^2^, which was used as the query for searching the deduced protein sequences using the HMMER search program^3^. The candidate protein sequences were submitted to the SMART database^4^ and NCBI-CCD website^5^ to verify the conserved domains of SOD proteins. The molecular weight and theoretical iso-electric point of NtSOD proteins were calculated using the ProtParam tool in ExPASy web^6^. The subcellular localization of NtSOD proteins was predicted using the BUSCA web server^7^ (Savojardo et al., 2018).

### Phylogenetic Analysis

Twenty-three SOD proteins were selected to construct an unrooted phylogenetic tree using MEGA 7 with the neighbor-joining (NJ) algorithm and 1,000 bootstrap replicates to investigate the phylogenetic relationships of the *SOD* genes between tobacco and *A. thaliana*; other parameters all used the default setting. Finally, the iTOL online tool^8^ was used to visualize the phylogenetic trees.

### Multiple Sequence Alignment, Motif Composition and Gene Structure

Multiple sequence alignment was performed with the MAFFT software (Katoh and Standley, 2013) using the FFT-NS-2 algorithm and subsequently visualized using Jalview software packages to construct consensus sequences of NtSOD family members (Waterhouse et al., 2009). An internal consistency analysis was conducted on each of the three groups of *NtSOD* genes using BioEdit software. Multiple EM for Motif Elicitation (MEME) suite (http://meme-suite) was used with the default parameters to determine the distribution of conserved motifs within the NtSOD proteins (Bailey et al., 2015). The diagrams of the exon-intron structure of *NtSOD* genes were generated using the online tool GSDS^9^ according to the available coding sequence and their respective genomic sequence (Hu et al., 2015). The composite picture of the phylogenetic tree, motif distribution and gene structure of *NtSOD* genes was generated using the Gene Structure View program of TBtools software (Chen et al., 2020).

### Prediction of *Cis*-Acting Elements

Two thousand-bp sequences upstream of the translation start site were extracted as promoter regions using the Gtf/Gff3 Sequences Extract program of TBtools software to further understand the potential functions of the *cis*-regulatory elements in *NtSOD* genes. The *cis*-regulatory elements in the promoter sequences were analyzed with the PlantCare online tool^10^, and then the results were visualized with the Simple BioSequence Viewer of TBtools software (Chen et al., 2020).

### Prediction of Transcription Factors and miRNAs Involved in Regulating NtSOD Expression

As a method to better understand the transcription factors and miRNAs involved in regulating *NtSOD* expression, the promoter and mRNA sequences were used to predict possible transcription factor binding sites and target miRNAs, respectively. In detail, 2000-bp promoter sequences of *NtSOD* genes were submitted to the PlantRegMap website^11^ to predict the transcription factors involved in regulating *NtSOD* expression with the Regulation Prediction tool at a *p* value ≤ 1e^–6^ (Tian et al., 2020). For the miRNA target gene analysis, NtSOD mRNA sequences were submitted to the psRNATarget online server^12^, and then the network map was generated using Cytoscape software (Dai et al., 2018).

### Plant Materials and Heavy Metal Treatments

Seeds of tobacco cultivar TN90 were sown in uncontaminated nutrient soil and generated plantlets in a greenhouse at 25 ± 2°C under a 16/8 h (light/dark) photoperiod. Two-week-old tobacco seedlings were transplanted to hydroponic tanks and cultured with half-strength Hoagland’s solution for 7 days. Heavy metal stress was induced by replacing the solution in hydroponic tanks with fresh Hoagland’s solution containing 200 μM CuSO_4_, 200 μM ZnSO_4_, 200 μM MnSO_4_, 200 μM Fe-EDTA, or 50 μM CdCl_2_. For ion-deficiency treatments, the solutions were replaced with fresh Hoagland’s solution lacking Cu, Zn, Mn and Fe (Yu et al., 2021). The tobacco seedlings were cultured with the abovementioned treatments for 7 days. Subsequently, the leaves and roots of the seedlings were separately harvested. For the analysis of the tissue expression profile, five tissue samples, including roots, stems, old leaves, young leaves and flowers, were collected from four-month-old tobacco plants grown in the natural environment. All samples were frozen immediately in liquid nitrogen and stored at −80°C until total RNA was isolated. Each experimental group consisted of three biological replicates and technical duplicates.

### Determination of the Heavy Metal Content in Tobacco Plants

The metal ion concentrations in the roots and shoots of tobacco plants treated with various heavy metals were measured in this study. The roots of tobacco plants were soaked in a 20 mM EDTA solution for half an hour and then washed three times with distilled water to remove heavy metal ions and precipitates adsorbed on the root surface with sterile absorbent paper for laboratory. The plant materials, including roots and shoots, were dried in an oven at 80°C for 3 days until a constant weight was obtained. Subsequently, the dried roots and shoots were separately ground into powders, and 20 mg of powdered sample was digested with 7 mL of concentrated HNO_3_ for 2 h at 115°C. The contents of Cd, Cu, Zn, Fe and Mn were determined using flame atomic absorption spectrometry (TAS-986, China) at wavelengths of 228.8 nm, 324.8 nm, 213.9 nm, 248.3 nm and 279.5 nm, respectively.

### Analysis of the Pattern of NtSOD Gene Expression

Total RNA was extracted from the abovementioned plant materials using an RNAsimple Total RNA kit (DP419, Tiangen Biotech, Beijing, China) according to the manual, and then the quality and concentration of RNA were determined using a BioPhotometer Plus instrument (Eppendorf, Germany). Subsequently, 5 μg of total RNA were used to synthesize first-strand cDNAs *via* reverse transcription using the GoScriptTM Reverse Transcriptase Kit (Promega, Madison, WI, United States) according to the manufacturer’s protocol. All cDNA samples were diluted 25 times with RNase-free water and stored at −20°C until qPCR was performed.

In the present study, *NtSOD* gene expression was analyzed based on transcriptome data and qPCR results. The raw transcriptome data (PRJNA208209) of tobacco cultivar TN90 were downloaded from NCBI-BioProject, including SRA data from roots, stems, young leaves, mature leaves, senescent leaves, young flowers, mature flowers, and senescent flowers. The relative abundance of each gene transcript was calculated as transcripts per kilobase million (TPM) values using the Salmon program and visualized in a heatmap using the Heatmap tool of TBtools. qPCR was performed with a CFX96TM real-time fluorescence quantification platform (Bio-Rad, United States) using SYBR Green enzyme (Novoprotein, China) with the following procedure to further verify the results of transcriptome data analysis: 95°C for 1 min, followed by 45 cycles of 95°C for 15 s and 60°C for 30 s. The qPCR primers were designed using Primer Premier 6.0 and are listed in Supplementary Table 1. The relative mRNA expression levels of *NtSOD* genes were normalized to *NtEF1*α (accession number: AF120093) (Liu et al., 2022), and relative fold changes were calculated using the 2^–ΔΔ*CT*^ method (Livak and Schmittgen, 2001).

### Heterologous Expression and Functional Verification of NtSOD Family Genes in Yeast

The CDSs of three NtSOD family members, *NtCSD1a*, *NtFSD1e* and *NtMSD1b*, were cloned by PCR with the primers listed in Supplementary Table 1 to verify the potential function of NtSOD proteins in scavenging ROS produced in response to heavy metal stress. The PCR fragment and pYES2 vector were digested using the restriction enzymes *Bam*HI *and Eco*RI (TaKaRa, Dalian, China) for 3 h at 37°C. Subsequently, the CDS of the three genes were inserted into the expression vector pYES2 using the DNA Ligation Kit Ver. 2.1 (TaKaRa, Dalian, China) and transformed into *E. coli* strain DH5α. The inserted sequences in the resulting recombinant plasmids, named pYES2:NtCSD1a, pYES2:NtFSD1e and pYES2:NtMSD1b, were verified by DNA sequencing (BGI, Shenzhen, China). The recombinant plasmids were amplified in DH5α cells and extracted according to the operation manual of a high purity plasmid extraction kit (Biomed, Beijing, China). The oxidation-sensitive yeast mutant strain Δ*yap1* (*MAT*α *ura3lys2 ade2 trp1 leu2 yap1:leu2*) was transformed with the three recombinant plasmids using the lithium acetate transformation protocol, and the empty plasmid pYES2 was used as the control (Kawai et al., 2010; Rodrigues-Pousada et al., 2019). Yeast cells were cultured in SD/-URA liquid medium until reaching the logarithmic phase (OD_600_ = 0.6) to assess the sensitivity of the cells to heavy metal-induced oxidative stress. The cultures were successively diluted 10 times from 1 to 10^–3^ and spotted onto SD plates containing 50 μM CdCl_2_, 2.5 mM CuSO_4_, 5 mM ZnSO_4_, 2.5 mM FeSO_4_, or 2.5 mM MnSO_4_ and grown for 3 days before being photographed.

### Statistical Analysis

Data are presented as the means ± SD derived from at least three biological replicates, unless indicated otherwise. All data were analyzed using one-way ANOVA, and significant differences were analyzed using Dunnett’s multiple range test (*P* < 0.05) with GraphPad Prism 8.0 (GraphPad Software, San Diego, CA, United States).

## Results

### Identification and Phylogenetic Analysis of NtSOD Genes

**FIGURE 1:**
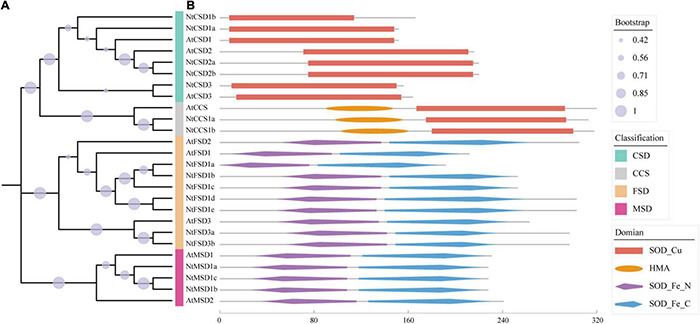
Phylogenetic analysis and distribution of conserved structural domains of SOD gene family members in *N. tabacum* and *Arabidopsis*. **(A)** Phylogenetic tree of AtSODs and NtSODs. **(B)** Distribution of conserved structural domains of AtSODs and NtSODs. Red squares indicate the SOD_Cu structural domain (PF00080), orange oval blocks indicate the HMA structural domain (PF00403), and purple and blue diamond blocks represent the SOD_Fe_N structural domain (PF00081) and SOD_Fe_C structural domain (PF02777), respectively.

According to the results of the HMM search and BlastP, 17 candidate genes were originally obtained from the Pfam SOD family in tobacco. Based on the domain analysis, 5 proteins were shown to have a Cu/Zn-SOD domain (PF00080), 7 proteins contained an Fe/Mn-SOD alpha-hairpin domain (PF00081), and 3 proteins contained an Fe/Mn-SOD C-terminal domain (PF02777). Additionally, 2 proteins contained both a Cu/Zn-SOD domain and an N-terminal heavy metal-associated domain (HMA; PF000403) (Figure 1B), which were considered copper chaperones for Cu/Zn superoxide dismutase in tobacco (NtCCS) due to their clustering with AtCCS in the phylogenetic tree (Figure 1A). CCS is essential for transporting Cu to SOD but has no dismutase activity (Cohu et al., 2009). After excluding 2 NtCCS members from 17 candidate proteins, we identified 15 NtSOD proteins in *N. tabacum* cultivar TN90.

A neighbor-joining phylogenetic rootless tree was constructed using SOD protein sequences from *A. thaliana* and tobacco to further clarify the evolutionary relationship of *SOD* genes in tobacco. All *NtSOD* genes were assigned specific names according to their phylogenetic relationships with *AtSOD* genes. The results of phylogenetic analysis showed that SOD family members in tobacco were classified into three subfamilies with high bootstrap values, including the Cu/Zn-SOD subfamily (NtCSD1a/1b, NtCSD2a/2b, and NtCSD3), Fe-SOD subfamily (NtFSD1a/1b/1c/1d/1e and NtFSD3a/3b) and Mn-SOD subfamily (NtMSD1a/1b/1c). The NtFSD subfamily exhibited a closer phylogenetic relationship with NtMSD than with NtFSD, potentially because NtFSD and NtMSD have the same conserved domains SOD_Fe_N and SOD_Fe_C (Figure 1B).

The protein sequences and physicochemical properties of NtSOD family members were characterized in the present study. The *NtSOD* gene CDSs ranged from 459 bp to 912 bp and encoded NtSOD proteins with lengths ranging from 153–304 amino acids. The molecular weight of the NtSOD proteins ranged from 15.2 kDa to 34.7 kDa, and the theoretical isoelectric points (pIs) ranged from 5.08 (NtCSD1b) to 8.82 (NtMSD1c). Additionally, the results for the predicted subcellular localization showed that SOD proteins in the same subfamily may be located in different organelles. Except for NtFSD1a that is located in the cytoplasm, other members in the NtFSD subfamily are located in chloroplasts. NtCSD2a/2b is located in chloroplasts, while NtCSD1a/1b/3 is located in the extracellular space. NtMSD1c is the only tobacco SOD protein located in mitochondria (Table 1).

**TABLE 1:** The data of 15 *NtSOD* genes identified in tobacco genome.

Gene Name	Gene ID	Length of protein (aa)	MW (kDa)	pI	Subcellular prediction
NtCSD1a	LOC107790449	153	15.20	5.47	Extracellular space
NtCSD1b	LOC107774639	167	17.75	5.08	Extracellular space
NtCSD2a	LOC107806960	221	22.54	5.95	Chloroplast thylakoid membrane
NtCSD2b	LOC107767528	221	22.57	6.23	Chloroplast thylakoid membrane
NtCSD3	LOC107826236	157	15.71	6.78	Extracellular space
NtFSD1a	LOC107767307	193	21.84	5.51	Cytoplasm
NtFSD1b	LOC107805394	254	28.34	8.60	Chloroplast thylakoid membrane
NtFSD1c	LOC107800004	254	28.38	7.90	Chloroplast thylakoid membrane
NtFSD1d	LOC107819573	304	34.78	6.03	Chloroplast outer membrane
NtFSD1e	LOC107832827	304	34.69	5.91	Chloroplast outer membrane
NtFSD3a	LOC107797648	298	33.81	7.68	Chloroplast outer membrane
NtFSD3b	LOC107820063	298	33.87	7.71	Chloroplast outer membrane
NtMSD1a	LOC107803567	229	25.59	8.40	Organelle membrane
NtMSD1b	LOC107829594	229	25.55	7.86	Organelle membrane
NtMSD1c	LOC107830263	229	25.49	8.82	Mitochondrion membrane

### Multiple Sequence Alignment of NtSOD Proteins

**FIGURE 2:**
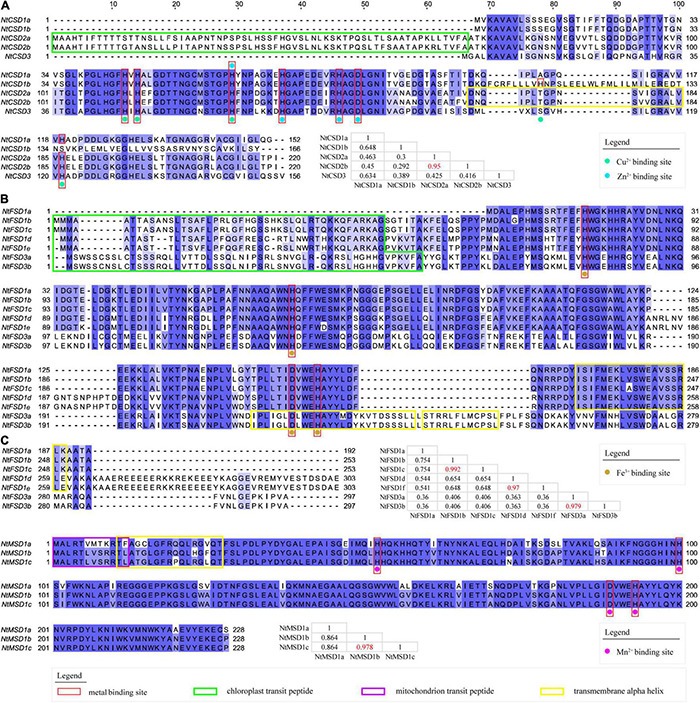
Multiple sequence alignment of deduced amino acid sequences of NtSOD proteins. **(A)** Multiple sequence alignment of NtCSD proteins. The metal-binding sites for Cu^2+^ and Zn^2+^ are marked with green and blue dots, respectively. **(B)** Multiple sequence alignment of NtFSD proteins. Metal-binding sites of Fe^3+^ are marked with brown dots. **(C)** Multiple sequence alignment of NtMSD proteins. Metal-binding sites of Mn^2+^ are marked with purple dots. The similarity between amino acid sequences was recorded in the table, and the chloroplast transit peptide, mitochondrial transit peptide, and membrane transit peptide are marked using green, purple and yellow boxes, respectively, and red boxes mark the metal-binding sites of these SOD proteins.

Multiple sequence alignment was conducted to analyze the differences among the fifteen NtSOD proteins, and the results are shown in Figure 2. Among the five members of the NtCSD subfamily, NtCSD2a and NtCSD2b had the highest sequence consistency of 95%. NtCSD1b showed low consistency with the other four subfamily members, ranging from 29.2% to 64.8%. The N-terminal and C-terminal regions of NtCSD2a/2b contained chloroplast transporter peptide sequences and transmembrane alpha-helical structures, respectively, suggesting that NtCSD2a/2b was localized in the chloroplast membrane. These results were consistent with the predicted subcellular localization of NtCSD2a/2b. Eight metal binding sites were identified in the protein sequence of the NtCSD gene subfamily, of which three sites bind Cu, four sites bind Zn, and H63 binds both Cu and Zn (Figure 2A). In the NtFSD subfamily, the sequence consistency between NtFSD1b and NtFSD1c, NtFSD1d and NtFSD1e, and NtFSD3a and NtFSD3b was greater than 97%, suggesting that they may be the products of gene doubling events. All NtFSD proteins (except NtFSD1a) have a chloroplast transit peptide and transmembrane alpha-helix, suggesting that these enzymes function on the chloroplast membrane. Although NtFSD1a has a transmembrane structure, the absence of its chloroplast transporter peptide leads to its localization in the cytoplasm, consistent with the predicted subcellular localization. In addition, the four Fe ion binding sites were highly conserved in all members of the NtFSD gene subfamily (Figure 2B). The three members of the NtMSD subfamily exhibit high sequence consistency, among which NtMSD1b and NtMSD1c have only 5 amino acid differences, and the similarity is 97.8%. The mitochondrial transit peptide and transmembrane alpha-helix are closely linked at the N-terminus of NtMSD, consistent with the prediction that NtMSD is located in mitochondria by the subcellular localization analysis. The four Mn binding sites are extremely conserved in NtMSD subfamily members (Figure 2C).

### Conserved Motifs and Gene Structure Analysis of NtSOD Genes

**FIGURE 3:**
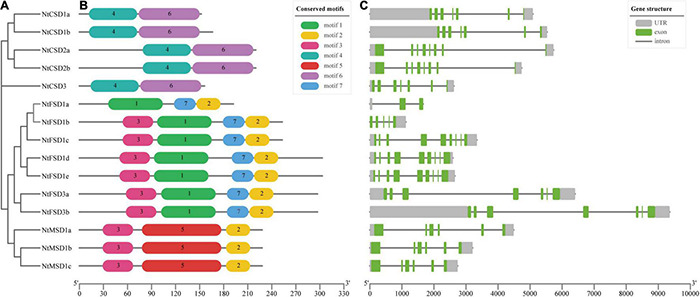
Phylogenetic tree, conserved motifs of NtSOD proteins, and exon-intron structures of *NtSOD* genes. **(A)** A neighbor-joining tree of NtSOD proteins. **(B)** Seven motifs in NtSOD proteins identified using the MEME tool are marked by different colors. **(C)** Exon-intron structures of *NtSOD* genes. Introns, UTRs, and CDSs are displayed with gray lines, gray boxes, and green boxes, respectively.

We further analyzed the conserved motifs of the NtSOD family genes in tobacco, and 7 motifs were identified using MEME software with the default parameters (Figure 3B). As expected, most of the closely related members in the same subfamily had common motif compositions; however, no common motifs were shared in all fifteen *NtSOD* genes. Among them, motifs 4 and 6 were associated with the Cu/Zn-SOD domain (PF00080) and were only identified in Cu/Zn-SOD subfamily members. Motif 1, motif 7 and motif 5 were specific to NtFSD and NtMSD proteins, respectively. Motif 2 and motif 3 correspond to the Fe/Mn-SOD alpha-hairpin domain (PF00081) and Fe/Mn-SOD C-terminal domain (PF02777), respectively. Except for NtFSD1a, motif 3 was the common conserved motif in the NtFSD subfamily. Motif2 was shared by the NtFSD subfamily and NtMSD subfamily.

The number of introns and gene structure are usually related to the evolution of gene family members (Zhang et al., 2021). In the present study, cDNA and corresponding DNA sequences of NtSOD family genes were compared to analyze the gene structure (Figure 3C). *NtFSD1b* was the smallest gene at 1,124 bp, while *NtFSD3b* was the longest gene at 9,355 bp. All *NtSOD* genes contain introns, and the number of introns ranges from 2 to 8. Among them, *NtFSD1a* has the least number of introns at 2, while *NtFSD1c/1d/1e* has the most at 8 introns. Notably, the three *NtFSD1c/1d/1e* genes showed highly similar genetic structures, and their cDNA and DNA sequences were highly consistent, suggesting that they may be the result of gene replication. The same replication event occurs in *NtCSD1a/1b* and *NtCSD2a/2b*.

### *Cis*-Acting Elements in the Promoter Regions of NtSOD Genes

**FIGURE 4:**
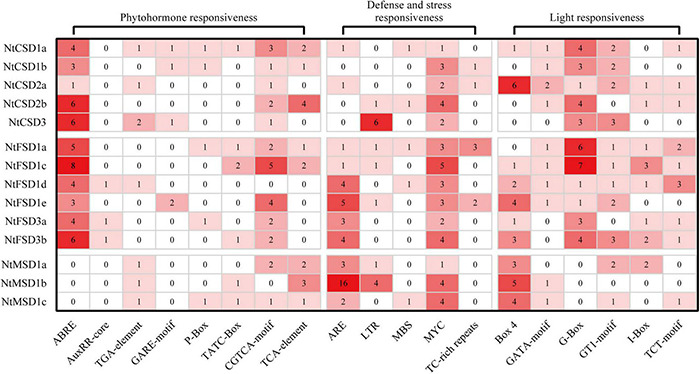
*Cis*-acting elements in the promoters of *NtSOD* genes. The frequencies of *cis*-acting elements are represented by numbers and different colors of shading.

Based on accumulating evidence, *SOD* genes play important roles in the plant response to abiotic stress. The *cis*-acting elements in the promoter regions of the NtSOD family genes, except *NtFSD1e* due to incomplete assembly of the tobacco genome sequence, were scanned using plantCARE to better understand the potential regulatory mechanism of *NtSOD* genes in abiotic stress or hormonal responses of tobacco. Nineteen elements involved in defense and stress responsiveness, phytohormone responsiveness and light responsiveness were detected in the *NtSOD* promoters and divided into three categories, as shown in Figure 4. These elements were irregularly dispersed in the promoter regions of NtSOD family genes (Supplementary Figure 3). No similar distribution pattern was observed between two genes, even those that are evolutionarily close. Many of the hormone-related *cis*-acting elements, including ABRE (abscisic acid), AuxRR core/TGA element (auxin), GARE motif/P Box/TATC Box (gibberellin), CGTCA motif/TGACG motif (MeJA), and TCA element (salicylic acid), were identified in the promoter region of *NtSOD* genes. ABRE and CGTCA/TGACG motifs were widely distributed in *NtFSD* and *NtCSD* promoter sequences, suggesting that ABA and MeJA are involved in the regulation of *NtFSD* and *NtCSD*. *NtMSD* was predicted to be regulated by auxin and salicylic acid based on the presence of TGA elements and TCA elements in their promoter regions, respectively. Elements associated with auxin and gibberellin responses are scattered in the promoters of NtSOD family genes (Figure 4). These results suggested that plant hormones might exert a modulatory effect on the regulation of *NtSOD* gene expression. MYC, one of the key motifs responding to thrilling, was identified in all NtSOD family gene promoter regions. The anaerobic induction regulation-related element ARE was mainly present in the promoters of NtFSD subfamily and NtMSD subfamily genes, especially in the promoter region of *NtMSD1b*, which contained 16 ARE elements. In addition, a large number of light-responsive elements were present in almost all promoter regions of *NtSOD* genes, among which Box4 and G-box were significantly enriched.

### Analysis of Potential Regulatory Interactions Between Transcription Factors and NtSODs

**FIGURE 5:**
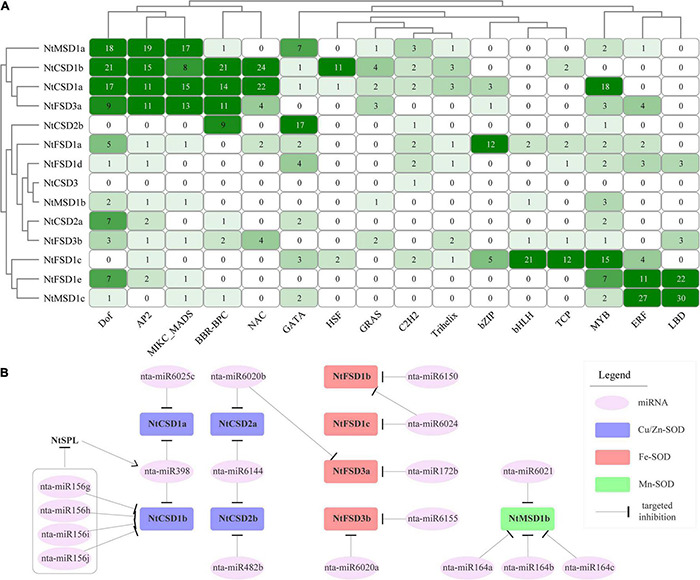
Predicted factors regulating the expression of *NtSOD* genes. **(A)** Transcription factor binding sites in the promoters of *NtSOD* genes. The frequencies of *cis*-acting elements are represented by numbers and different colors of shading. **(B)** The predicted miRNAs regulating the expression of *NtSOD* genes.

The online software PlantRegMap was employed to predict the potential regulatory interactions between transcription factors (TFs) and NtSOD family genes. Sixteen TFs that may be involved in regulating NtSOD family gene expression were identified. The number of potential binding motifs for TFs in NtSOD family gene promoters was counted to conduct a clustering analysis and construct a heatmap (Figure 5A). The regulatory patterns of *NtMSD1a*, *NtCSD1a*, *NtCSD1b* and *NtFSD3a* were similar, all of which were regulated by Dof, AP2 and MIKC_MADS. Additionally, three *NtSOD* genes, *NtFSD1c*, *NtFSD1e* and *NtMSD1c*, constitute another group with similar regulatory patterns, which were all regulated by MYB, ERF and LBD. Notably, the *NtCSD3* promoter only contains one C2H2 transcription factor binding site, suggesting that *NtCSD3* may not be regulated by transcription factors other than C2H2.

### Analysis of miRNAs Targeting NtSOD Genes

MicroRNAs (miRNAs) are a class of single-stranded small (20–24 nucleotides) non-coding RNAs that regulate gene expression by binding to target gene transcripts to inhibit their translation or promote mRNA degradation. psRNATarget online software was used to analyze miRNA binding sites in 15 *NtSOD* genes and to provide insights into the miRNA-mediated regulation of the *NtSOD* genes. Nine *NtSOD* genes were predicted to be targeted by 13 known miRNA families, of which 5 target genes were cleaved by 5 conserved miRNA families, including miR156, miR398, miR482, miR172 and miR164, and 7 target genes were cleaved by unique miRNAs in *Solanaceae* plants, including miR6025, miR6020, miR6144, miR6150, miR6024, miR6155 and miR6021 (Figure 5B). As expected, miR398 was predicted to target *NtCSD1a/1b*. The miR164 family and miR6021 may be involved in regulating the expression of *NtMSD1b*, which was the only gene regulated by miRNAs in the NtMSD subfamily of genes. Based on accumulating evidence, miR156-SPL and miR164-NAC modules regulate plant abiotic stress tolerance by maintaining ROS homeostasis (Fang et al., 2014; Yin et al., 2019). Interestingly, miR156 and miR164 were associated with the expression of *NtCSD1b* and *NtMSD1b* in tobacco, respectively. Therefore, the regulation of NtSOD family genes in tobacco involves a complex regulatory network of transcription factors and miRNAs.

### Tissue-Specific Expression Profiles of NtSOD Family Genes

**FIGURE 6:**
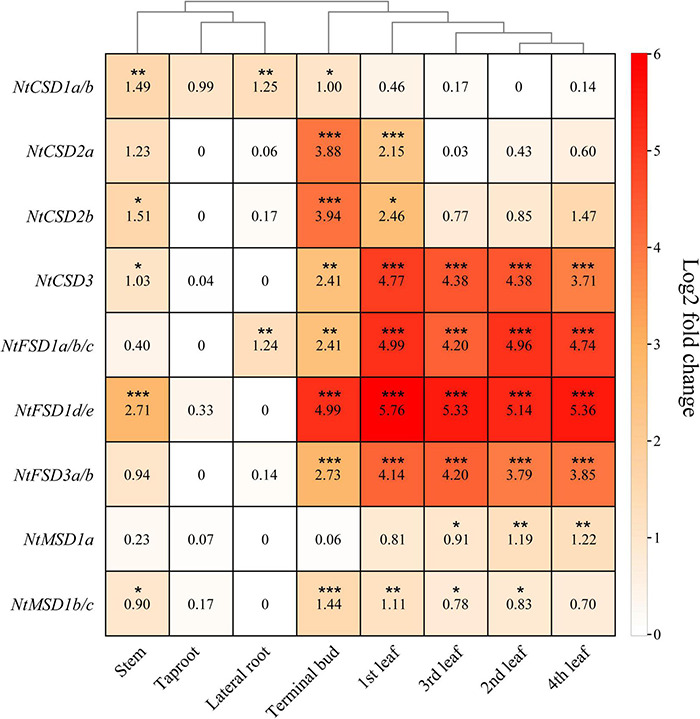
The relative expression levels of *NtSOD* genes in different tobacco tissues. The expression data were obtained from the real-time RT-PCR (RT-qPCR) analysis data and are shown as log2 values calculated as averages. High expression levels are shown in red, and lower expression levels are shown in white. Statistically significant differences are indicated using asterisks (Dunnett’s test, *p < 0.05, **p < 0.01, and ***p < 0.001). Data are presented as the means ± SD of three replicates.

qPCR was used to analyze the relative tissue-specific expression levels of NtSOD family genes in different organs (stem, taproot, lateral root, terminal bud, 1st leaf, 2nd leaf, 3rd leaf and 4th leaf) at the vegetative stage of tobacco grown under normal growth conditions. Taken together, the expression levels of NtSOD family genes in taproots and lateral roots were lower than those in shoots of tobacco plants. Similar expression patterns were observed among *NtSOD* genes in the same subfamily, which were hierarchically clustered in the heatmap (Figure 6). Additionally, the qPCR results were generally consistent with the results from the transcriptome data (Supplementary Figure 2). NtCSD subfamily genes, except *NtCSD3*, were expressed at relatively high levels in terminal buds and the 1st leaves but expressed at low levels in mature leaves. NtFSD subfamily genes shared a similar expression pattern and exhibited higher expression levels in leaves than in roots, while *NtFSD1d/1e* was expressed at high levels in stems. A lower expression level of NtMSD subfamily genes was detected in lateral roots, and no significant difference was observed in the other tissues.

### Heavy Metal Stress-Induced Expression Profiles of NtSOD Family Genes

**FIGURE 7:**
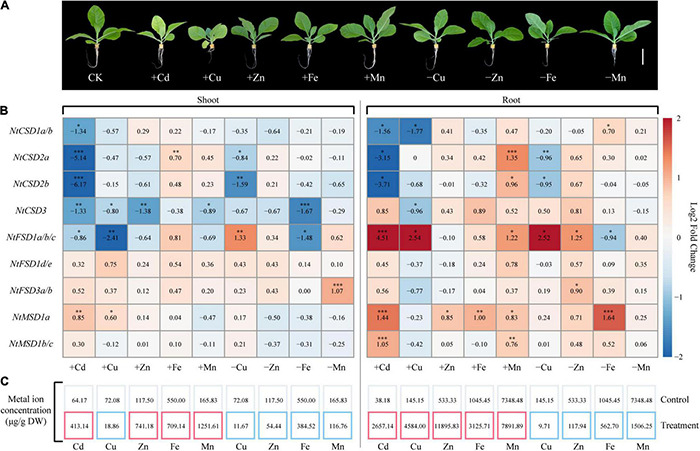
Expression of *NtSOD* genes in tobacco shoots and roots treated with Cd, Cu, Zn, Fe, or Mn stress and without Cu, Zn, Fe, or Mn. **(A)** The phenotypes of tobacco seedlings subjected to different heavy metal treatments. **(B)** qRT-PCR analysis of *NtSOD* gene expression in response to various heavy metal stresses. The numbers in the box represent the average ratio of gene expression levels in the treatment and control groups. The asterisks on the number indicate significant differences (Dunnett’s test, *p < 0.05, **p < 0.01, and ***p < 0.001). The population size was *n* = 3. **(C)** Content of metals contained in tobacco tissues subjected to different heavy metal treatments. The red box indicates that the corresponding metal content is higher than that of the control, while the blue box indicates that the corresponding metal content is lower than that of the control.

Two-week-old tobacco seedlings were divided into two treatment groups, including a heavy metal toxicity group (hydroponic solution containing Cd or excess Cu, Zn, Fe or Mn) and an ion-deficiency treatment group (Hoagland’s solution without Cu, Zn, Fe or Mn), to understand the effect of heavy metal stress on the expression pattern of NtSOD family genes. After 7 days of treatment, chlorosis and growth inhibition were observed as significant toxicity symptoms for tobacco seedlings treated with Cd and Cu. Additionally, the growth of roots of tobacco seedlings under Cu stress was substantially inhibited (Figure 7A). In the present study, the ion concentrations in all the treated tobacco seedlings were determined using flame atomic absorption spectrometry. The results were consistent with the expectation. In the ion-deficient stress groups, the ion contents in the roots and shoots of tobacco seedlings were significantly lower than those in the control group. In contrast, the ion content was higher in the heavy metal toxicity group than that in the control group (Figure 7C). Notably, the copper ions mainly accumulated in the roots of tobacco after treatment with 200 μM Cu, while the concentration of copper ions in the shoots of Cu-treated tobacco was lower than that in the control group. This phenomenon may be due to Cu stress inhibiting the growth and development of roots in tobacco, which blocked ion transport from roots to shoots. Thus, heavy metal stress disrupted ion homeostasis in tobacco plants in the present study.

Antioxidant enzymes, especially SOD, play important roles in scavenging ROS generated in response to heavy metal stress in plants. qPCR was used to analyze the relative expression levels of NtSOD family genes in tobacco seedlings with heavy metal toxicity and ion deficiency to clarify the expression patterns of NtSOD family genes in tobacco in response to oxidative stress induced by different heavy metals. Cd exposure significantly inhibited the expression of NtCSD subfamily genes and induced the upregulation of NtMSD subfamily genes (Figure 7B). The expression levels of *NtFSD1a/1b/1c* were significantly altered in the tobacco seedlings under Cu-related stress compared with the control group. However, the expression levels of *NtFSD1d/1e* were not affected by Cu toxicity or Cu deficiency. *NtCSD3* expression was downregulated in shoots but not in roots exposed to zinc toxicity. Except for *NtCSD2a* and *NtMSD1a*, iron toxicity had no effect on the expression of other NtSOD family genes. On the other hand, iron-deficient stress resulted in the downregulation of *NtCSD3* and *NtFSD1a/1b/1c* expression in shoots and significantly upregulated *NtMSD1a* expression in roots. Excessive accumulation of Mn in roots upregulated the expression of NtSOD family genes, including *NtCSD2a*, *NtMSD1b/1c*, *NtFSD1a/1b/1c*, *NtCSD2b* and *NtMSD1a*. However, Mn-deficient stress only induced the upregulation of *NtFSD3a/3b* expression in shoots. In summary, NtSOD family genes displayed a wide variety of expression patterns in tobacco seedlings in response to heavy metal toxicity and ion-deficient stresses.

### Heterologous Expression of *NtSOD* Genes in Yeast Mutant Δ*yap1*

**FIGURE 8:**
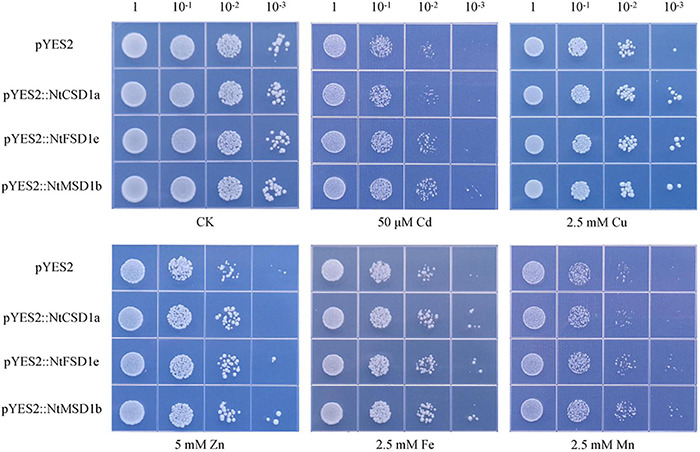
Effect of *NtSOD* gene expression in oxidation-sensitive yeast Δ*yap1* on yeast cell growth in response to different heavy metal stresses (50 μM Cd, 2.5 mM Cu, 5 mM Zn, 2.5 mM Fe, and 2.5 mM Mn). The numbers above the data represent the dilution times of the bacterial solution.

The oxidative stress-hypersensitive yeast mutant Δ*yap1* was transformed with *NtCSD1a*, *NtFSD1e* and *NtMSD1b* and compared with the control to investigate the ability of NtSOD family genes to resist oxidative stress induced by heavy metals. Four serial dilutions (10 ×) of yeast cells were dropped on solid SD/-URA medium containing different heavy metals, including Cd, Zn and Mn, and cultured for 3 days at 30°C. The dilution dot assay showed no significant differences in cell growth under normal culture conditions following the heterologous expression of three *NtSOD* genes in yeast and the control group. The Cd and Zn tolerance in *NtMSD1b*-overexpressing yeast was stronger than that in the control group. All yeast cells expressing *NtSOD* genes exhibited better growth than the control under Cu stress and Fe stress. Additionally, the heterologous expression of *NtMSD1b* or *NtFSD1e*, but not *NtCSD1a*, conferred tolerance to high concentrations of Mn (Figure 8). Based on these results, the overexpression of *NtSOD* genes in the mutant Δ*yap1* yeast effectively enhanced the resistance of yeast to heavy metal stress.

## Discussion

Tobacco is the most widely cultivated non-food cash crop in the world. ROS accumulation caused by stresses such as drought, high temperature and heavy metals exerts adverse effects on tobacco growth and development (Rizhsky et al., 2002). SOD is the first line of defense against oxidative stress and removes ROS that accumulate in plant cells under stress (Alscher et al., 2002). Previous studies have shown that SOD overexpression confers abiotic stress resistance to tobacco (Pitcher et al., 1991; Zhang et al., 2017; Pan et al., 2019). To date, studies on SOD family genes in tobacco have been limited. A complete characterization of the characteristics and functions of the SOD gene family in tobacco is helpful to enrich our understanding of the SOD gene family in plants. The systematic identification of the *NtSOD* genes as the best candidate genes for screening and enhancing tobacco heavy metal toxicity tolerance is very important to cope with the increasingly serious soil heavy metal pollution.

In this study, 15 *NtSOD* genes were identified from the tobacco genome and classified into three subfamilies according to their domains and motifs, including 5 *NtCSDs*, 7 *NtFSDs* and 3 *NtMSDs* (Table 1). Although NtCCS has a conserved SOD_Cu domain (Figure 1), its HMA domain and evolutionary relationship closely with *A. thaliana* suggested that NtCCS may be a copper chaperone for NtCSD. The N-terminus of the NtCCS protein contains a conserved metal-binding motif MxCxxC, consistent with AtCCS and MaCCS sequences (Chu et al., 2005; Leitch et al., 2009). In previous studies, the CCS gene has been classified as a member of the SOD family (Feng et al., 2016). Since NtCCS lacks the common motif of the NtCSD subfamily, NtCCS does not belong to the NtSOD gene family, and the NtCCS subfamily was not included in the subsequent gene family analysis. Additionally, the NtFSD2 subfamily is lacking in tobacco (Figure 1). Just like the NtMT family genes (Yu et al., 2021), The NtFSD2 subfamily gene loss events possibly occurred during the formation of tobacco tetraploid genome.

The phylogenetic analysis showed that NtFSDs and NtMSDs were clustered into one group with a high bootstrap value (Figure 3A). In addition, an analysis of conserved domains showed that NtFSD and NtMSD shared Motif 3 and Motif 7, while the conserved domains in the NtCSD subfamily were Motif 4 and Motif 6 (Figure 3B). These results suggest different origins of NtCSD and NtFSD/NtMSD. The emergence of SOD family enzymes is an important event of biological evolution on earth. Before the Great Oxidation Event, Fe and Mn were relatively abundant and highly available in the early reductive ocean, while Zn and Cu were bound to unavailable sulfur-bearing minerals in the crust (Saito et al., 2003). Thus, FSD and MSD were generally considered more ancient than CSD and evolved from a common ancestor (Wang et al., 2017). CSD, however, evolved separately in bryophytes. These two groups evolved independently. In addition, according to the distribution characteristics of motifs, we speculated that NtFSD1a might be an intermediate transition type between NtFSDs and NtMSDs (Figure 3B).

The NtCSDs were mainly localized in the cytoplasm (NtCSD1a/1b) and chloroplasts (NtCSD2a/2b) (Table 1), and exhibit low amino acid sequence identity (Figure 2). The gene structure analysis revealed significant differences in the number and location of introns between NtCSD1a/1b and NtCSD2a/2b (Figure 3), which is a feature that distinguishes cytoplasmic and chloroplast CSDs. The cytoplasmic CSD in *Marchantia paleacea* shares high homology with the chloroplast CSD, and MaCSD is speculated to be the closest relative to the original ancestral plant CSD (Fink and Scandalios, 2002). Unlike other NtFSD subfamily members, NtFSD1a is predicted to be expressed in the cytoplasm. Actually, FSDs were also expressed in the cytoplasm of cowpea and *L. japonicus* (Moran et al., 2003; Rubio et al., 2007). Plant MSDs are generally located in the mitochondria of plants, where NtMSD1c is predicted to be localized in the mitochondria. In addition, MSDs are expressed in peroxisomes (del Río et al., 2003), which explains the prediction that NtMSD1a/1b is expressed in organelle membranes (Table 1).

The analysis of *cis*-acting elements in promoters provides important information for studying the regulation of SOD expression. Due to the lack of NtFSD1b promoter information in the tobacco genome database, we extracted 14 *NtSOD* gene promoter sequences and performed a *cis*-acting element analysis. A large number of *cis*-acting elements related to plant hormone response existed in the promoter region of NtSOD family genes (Figure 4), which was consistent with the fact that SOD was regulated by ABA and MeJA in plants (Agarwal et al., 2005; Cao et al., 2009; Lu et al., 2009; Jiang et al., 2015). Plant SODs play an important role in defense and stress responses. Drought, low temperature, heavy metal toxicity, and biotic stresses usually lead to an imbalance in ROS homeostasis, which induces the expression of plant *SOD* genes. We identified multiple *cis*-acting elements associated with defense and stress resistance in the *NtSOD* gene promoter region (Figure 4). MYC and LRT, two *cis*-acting elements associated with freezing injury and low-temperature stress, were distributed in each *NtSOD* gene promoter region, a prediction that suggests an important role for the tobacco NtSOD family in responding to low-temperature stress. Overexpression of the *NtMSD* gene in alfalfa significantly enhanced the cold tolerance and next-year yield of transgenic plants (McKersie et al., 1993). ARE, a key *cis*-acting element involved in anaerobic induction, is widely distributed in the promoter region of the *NtSOD* gene, which might explain why SOD activity is increased in plants under hypoxic conditions (Chen and Qualls, 2003). *NtSOD* gene transcripts were significantly increased under light conditions, and conversely, *NtSOD* gene expression was significantly downregulated in the absence of light (Tsang et al., 1991). This phenomenon is consistent with our finding of a large number of *cis*-acting elements associated with light response in the tobacco *SOD* gene promoter. The analysis of *cis*-acting elements in the promoter indicated that *NtSOD* genes play an important role in the tobacco response to stress resistance.

The expression of *SOD* genes in response to stress is regulated by transcription factors and miRNAs in plants. *SOD* gene expression levels were significantly upregulated in transgenic peanut overexpressing *MuWRKY3*, which subsequently effectively enhanced drought stress tolerance in transgenic plants (Kiranmai et al., 2018). The transcription factor SPL7 is the main transcription factor that regulates *CSD* genes and negatively regulates the expression of *CSD* genes to adapt to Cu deficiency stress (Mermod et al., 2019). Activation of rat *SOD* gene expression by ginsenoside Rb2 from a ginseng extract is regulated by the AP2 transcription factor (Kim et al., 1996). Expression of the transcription factor NAC derived from maize (*Glycine max*) in tobacco resulted in transgenic plants that were more sensitive to drought, and the molecular mechanism was that NAC functioned as a negative regulator, reducing the expression of plant *SOD* genes and leading to ROS accumulation (Jin et al., 2013). Multiple transcription factor binding sites were predicted in the promoter region of the *NtSOD* genes, including Dof, AP2, NAC, MADS, and MYB (Figure 5). This result implies that *NtSOD* genes have different regulatory modalities to cope with the complex external environment. In recent years, an increasing number of studies have shown that miRNAs play a key role in the posttranscriptional regulation of plant *SOD* genes. miRNA398 is the first miRNA identified to negatively regulate *CSD* expression and plays an important role in plant resistance to stress. In *A. thaliana*, oxidative stress does not directly regulate the expression of *CSD* genes but represses miRNA398 expression, thereby protecting CSD mRNAs from cleavage (Zhou et al., 2019).

Plant *SOD* genes show different expression patterns at different growth stages or in different tissues. Some *SOD* genes exhibit constitutive expression in all tissues (Lin and Lai, 2013), while other *SOD* genes show tissue-specific expression patterns (Feng et al., 2015). In the present study, we detected the expression patterns of *NtSOD* genes in different tissues and at different stages using qPCR and nine-pair primers. The results of the qPCR analysis (Figure 6) were consistent with the results obtained from transcriptomic data (Supplementary Figure 2). Furthermore, *NtSOD* gene expression profiles showed that genes in the same subfamily have similar expression patterns, consistent with the tissue expression profile of cotton SOD family genes (Wang et al., 2017). Differences in the expression levels of NtSOD family members were observed in different tissues and leaves at different developmental stages. For example, the higher expression level of *NtFSD1a/b/c* in the lateral root, compared to other *NtSOD* genes, implies that it is associated with the development of the lateral root. The *fsd1* knockout mutant in *Arabidopsis* significantly suppressed the development of the lateral roots (Dvořák et al., 2021). *NtSOD* was expressed at a relatively high level in the terminal bud of tobacco, consistent with the continuous increase in SOD activity observed in buds of *Ficus carica* during the dormancy to germination period (Sedaghat et al., 2022). Briefly, the expression of tobacco NtSOD family genes show spatiotemporal specificity and play an important role in different tissues and organs and developmental stages.

The regulation of plant *SOD* gene expression by heavy metals is mainly reflected in two aspects. First, the toxic effects of heavy metal stress on cells induce the accumulation of ROS and a subsequent imbalance of cellular redox homeostasis; ROS removal usually depends on the expression of SOD. Second, metals such as Fe, Cu, Mn and Zn are essential to ensure SOD activity, and their deficiency also induces SOD expression. SOD expression levels in plants are usually upregulated by Cd, which is a non-essential and poisonous heavy metal (Pan et al., 2019). In contrast to our expectations, we found that Cd stress caused a significant decrease in the expression levels of NtCSD subfamily genes, consistent with the observation that Cd treatment exerted a significant inhibitory effect on the CSD mRNA and protein levels and activity in pea (Romero-Puertas et al., 2007). However, Cd stress significantly increased the expression levels of *NtFSD1a/b/c* and *NtMSD1a/b/c* (Figure 7), indicating that NtSOD family genes employ a differential division of labor in resisting Cd stress. Compared to toxicity induced by other essential metals, Mn toxicity significantly altered the expression level of *NtSOD* genes, especially in tobacco roots (Figure 7). A significant increase in SOD activity was detected in soybean roots treated with excess Mn (Santos et al., 2017). The SPL7-miR398 module is an important regulator of Cu homeostasis in *Arabidopsis*. Cu deficiency induces miR398 expression and results in degradation of the CSD mRNA (Yamasaki et al., 2009). The expression level of the NtCSD subfamily in tobacco was significantly downregulated under Cu-deficient conditions, suggesting that SPL7-mir398 is involved in a conserved regulatory module in plants to address disrupted Cu homeostasis. In addition, Cu deficiency induced the expression of *NtFSD1a/b/c* to complement the functional deficiency of *NtCSD*. Fe deficiency downregulated *NtFSD* expression but induced the expression of the *NtMSD* genes, consistent with the upregulation of MSD expression in *Chlamydomonas reinhardtii* under Fe deficiency (Allen et al., 2007). Consequently, *NtSOD* expression is regulated by a complex regulatory network to counteract the imbalance of heavy metal homeostasis in tobacco.

A yeast (*Saccharomyces cerevisiae*) mutant strain was used in this study to analyze the biological functions of NtSOD in resisting oxidative stress induced by heavy metal exposure. Yeast is generally acknowledged as a well-understood eukaryote in the field of stress response. The yeast activator protein (YAP) protein family is the largest bZIP subfamily in *S. cerevisiae* and consists of 8 members (Yap1 to Yap8) (Rodrigues-Pousada et al., 2019). In yeast cells, functional deficiency of Yap1 caused cellular sensitivity to ROS generated by the disruption of metal homeostasis (Schnell et al., 1992). Therefore, utilization of the yeast mutant strain delta yap represents a convenient method to test the ability of heterologously expressed SOD to remove ROS in response to heavy metal stress. In the present study, three tobacco *SOD* genes, *NtCSD1a*, *NtFSD1e* and *NtMSD1b*, were introduced into the yeast mutant strain Δ*yap1*. The results of the dilution dot assay showed that all three tobacco *SOD* genes alleviated oxidative stress induced by heavy metals (Figure 8). Compared to *NtMSD1b* and *NtFSD1e*, *NtCSD1a* may have a slightly weaker ability to remove ROS induced by heavy metals. Thus, *NtSOD* genes show differences in their abilities to remove ROS generated in response to heavy metals.

## Conclusion

In the present study, 15 *SOD* genes were identified in tobacco variety TN90. The NtSOD gene family is divided into three subfamilies: NtCSD, NtFSD and NtMSD. The specific characteristics of the *SOD* genes were investigated, including the subcellular localization, conserved domain and gene structure. *Cis*-regulatory elements of the *NtSOD* gene promoter region in response to plant hormones, abiotic stress and light were also identified. Meanwhile, the transcription factors and miRNAs that may be involved in regulating *NtSOD* gene expression were predicted. The analysis of the tissue expression profile showed that the NtSOD gene family played an important role in tobacco during the growth and development stages. The expression pattern of the NtSOD gene family in response to heavy metals indicated that the *NtSOD* genes were the key proteins mediating tobacco resistance to heavy metal toxicity, but their functions were different. In addition, the biological function of the NtSOD gene family in protecting against oxidative stress induced by heavy metals was verified in the yeast mutant strain. These results provide a good understanding of the biological characteristics and functions of the NtSOD gene family in tobacco and provide important information for the analysis of tobacco resistance to heavy metal-induced oxidative stress.

## Data Availability Statement

The original contributions presented in the study are included in the article/Supplementary Material, further inquiries can be directed to the corresponding author/s.

## Author Contributions

CH: data curation, methodology, and writing-original draft. CH and LH: investigation. LH: software. TY and SZ: methodology. RL and XJ: validation. WL, RL, and HX: funding acquisition. WL and FZ: writing-review. HX: resources. WL: conceptualization, editing, supervision, and project administration. All authors contributed to the article and approved the submitted version.

## Conflict of Interest

The authors declare that the research was conducted in the absence of any commercial or financial relationships that could be construed as a potential conflict of interest.

## Publisher’s Note

All claims expressed in this article are solely those of the authors and do not necessarily represent those of their affiliated organizations, or those of the publisher, the editors and the reviewers. Any product that may be evaluated in this article, or claim that may be made by its manufacturer, is not guaranteed or endorsed by the publisher.

## Footnotes

1
https://www.ncbi.nlm.nih.gov/genome/425?genome_assembly_id=274804

2
https://pfam.xfam.org/

3
http://hmmer.org

4
http://smart.embl.de/

5
https://www.ncbi.nlm.nih.gov/Structure/bwrpsb/bwrpsb.cgi

6
http://www.expasy.org/

7
http://busca.biocomp.unibo.it/

8
https://itol.embl.de/

9
http://gsds.gao-lab.org/

10
http://bioinformatics.psb.ugent.be/webtools/plantcare/html/

11
http://plantregmap.gao-lab.org/regulation_prediction.php

12
http://plantgrn.noble.org/psRNATarget/

## Supplementary Material

Supplementary Figure 1 Conserved motifs of NtSODs. The number on the X-axis indicates the position of the amino acid, and the number on the Y-axis indicates the conservation of the amino acid in the protein. The height of a letter indicates its relative frequency at the given position (X-axis) in the motif.

Supplementary Figure 2 Expression patterns of the tobacco *NtSOD* genes in various tissues. Heatmap generated with TPM values. The depth of the red color in the figure reflects the levels of gene expression.

Supplementary Figure 3 Distribution of *cis*-acting element positions in *NtSOD* promoters.
