# Supplementary figures and images for: The Superoxide Dismutase Gene Family in Nicotiana tabacum: Genome-Wide Identification, Characterization, Expression Profiling and Functional Analysis in Response to Heavy Metal Stress
Source: Front Plant Sci. 2022 May 6;13:904105. doi: 10.3389/fpls.2022.904105 (PMC9121019; doi:10.3389/fpls.2022.904105)

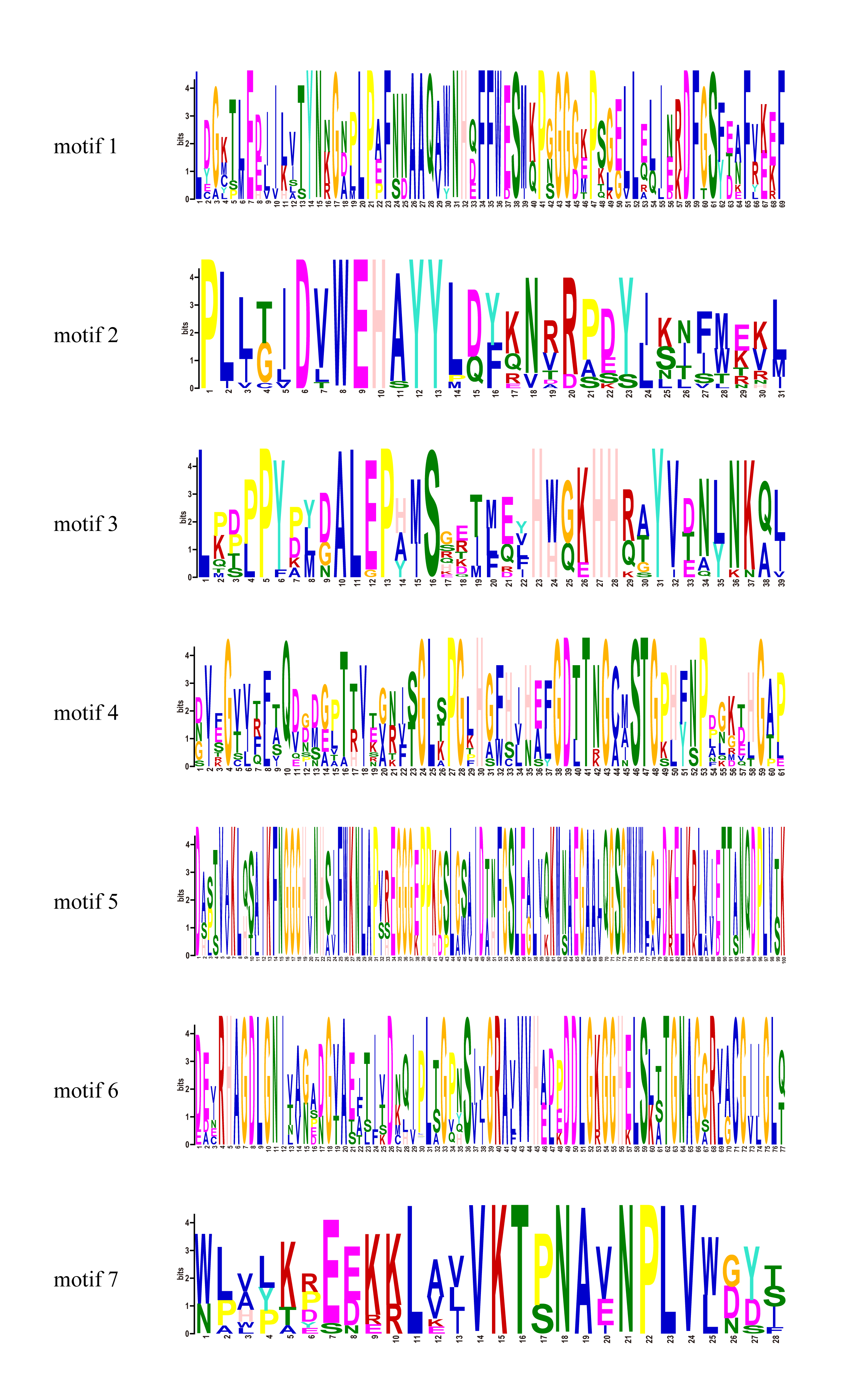

Supplement: Supplementary Figure 1 — Conserved motifs of NtSODs. The number on the X-axis indicates the position of the amino acid, and the number on the Y-axis indicates the conservation of the amino acid in the protein. The height of a letter indicates its relative frequency at the given position (X-axis) in the motif. [file Image_1.TIF]

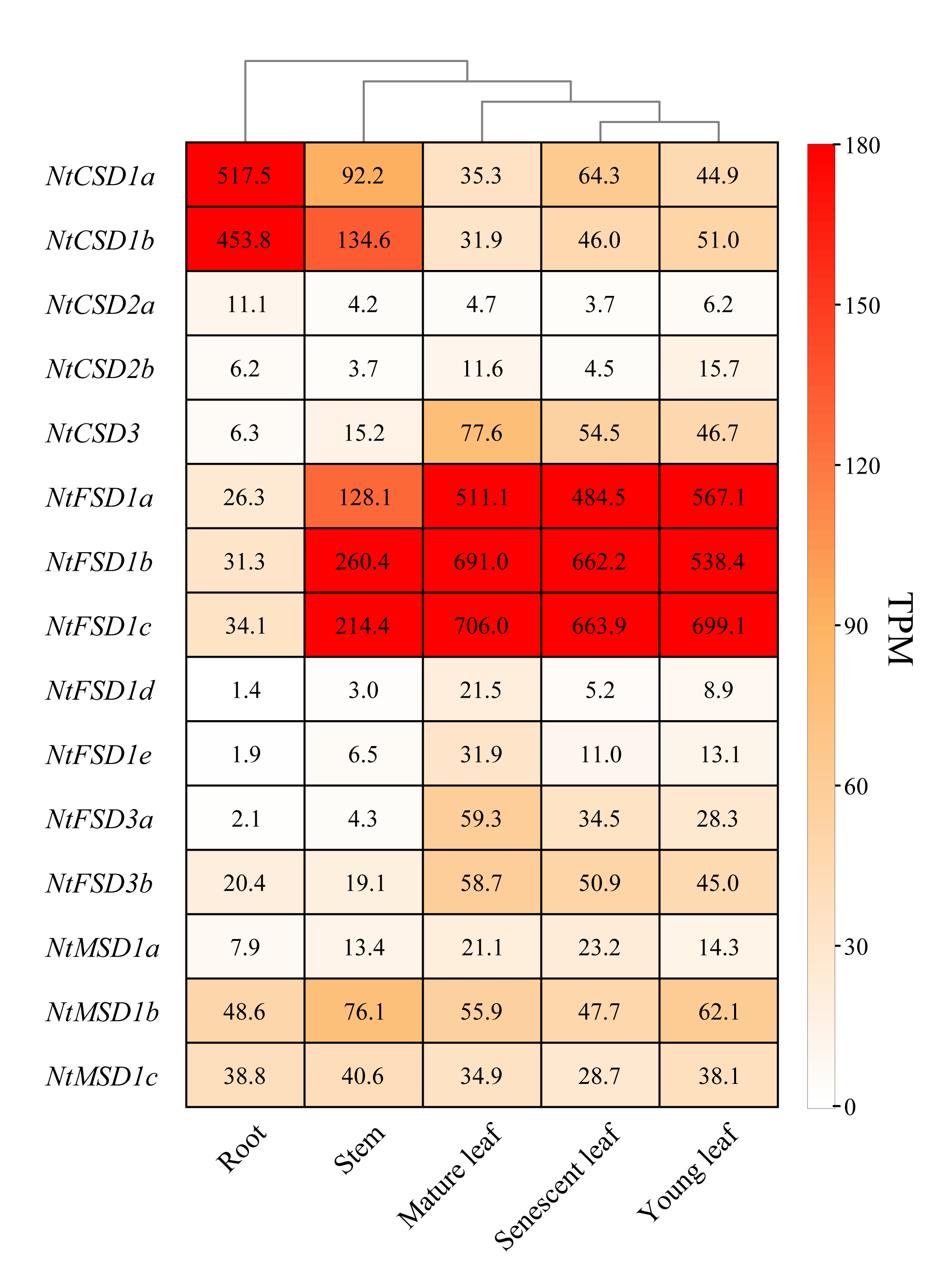

Supplement: Supplementary Figure 2 — Expression patterns of the tobacco NtSOD genes in various tissues. Heatmap generated with TPM values. The depth of the red color in the figure reflects the levels of gene expression. [file Image_2.TIF]

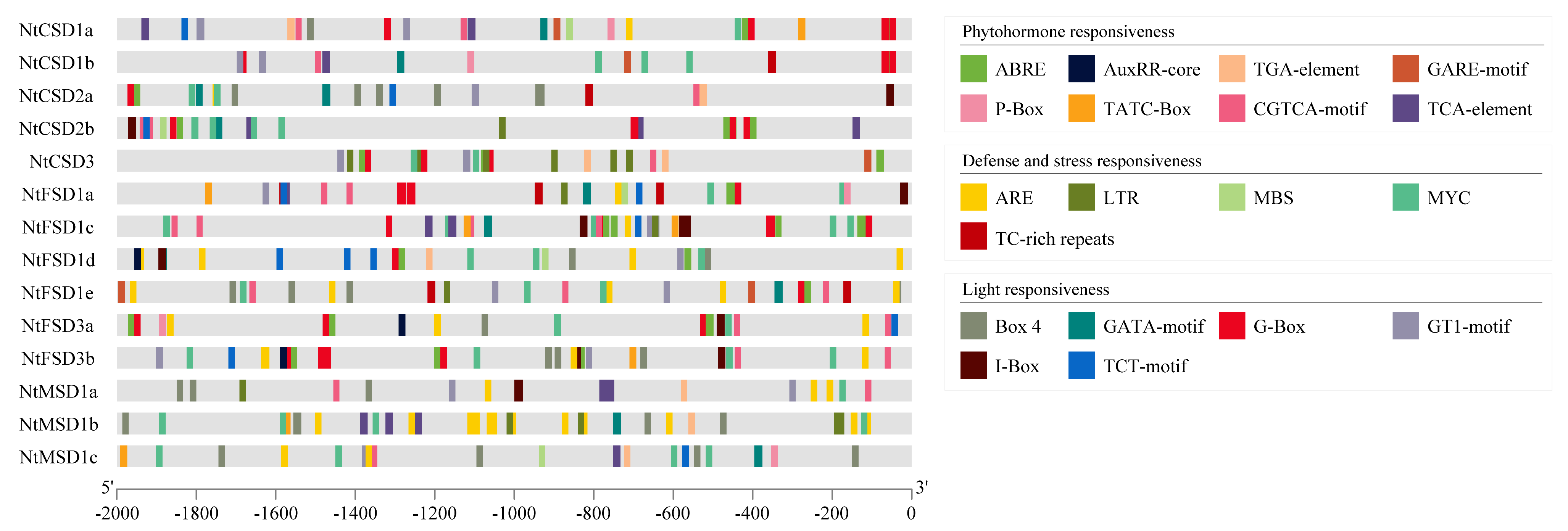

Supplement: Supplementary Figure 3 — Distribution of cis-acting element positions in NtSOD promoters. [file Image_3.TIF]
