# Supplementary material for: The Superoxide Dismutase Gene Family in Nicotiana tabacum: Genome-Wide Identification, Characterization, Expression Profiling and Functional Analysis in Response to Heavy Metal Stress
Source: Front Plant Sci. 2022 May 6;13:904105. doi: 10.3389/fpls.2022.904105 (PMC9121019; doi:10.3389/fpls.2022.904105)
Supplement: Supplementary file 4 [file Table_1.DOCX]

**Table S1. The primers used for the present study**

| Primer name | Gene symbol | Primer sequences (5’-3’) | Length of product (bp) |
| --- | --- | --- | --- |
| qF-NtEF1α | NtEF1α | TGAGATGCACCACGAAGCTC | 51 |
| qR-NtEF1α |  | CCAACATTGTCACCAGGAAGTG |  |
| qF-NtCSD1 | NtCSD1a/b | CCACAATCCATCATTGGAAGAGC | 125 |
| qR-NtCSD1 |  | CCGATGATACCACAAGCAAC |  |
| qF-NtCSD2a | NtCSD2a | GCCATGAACTCAGCCTTACCAC | 138 |
| qR-NtCSD2a |  | AGAGCTTTATTCCGAACCTGGG |  |
| qF-NtCSD2b | NtCSD2b | ACTCAGTTGTTGGAAGAGCACT | 130 |
| qR-NtCSD2b |  | GGAGTCAAACCAAGTATGCCAC |  |
| qF-NtCSD3 | NtCSD3 | TGGGAAGAGGTGGACATGAACT | 139 |
| qR-NtCSD3 |  | ACGAGTACTTTGACAGGAGCAA |  |
| qF-NtFSD1a/b/c | NtFSD1a/b/c | CACACCGCTCCTCACCATAGAC | 126 |
| qR-NtFSD1a/b/c |  | AGCCTAGAACTGACTGCTTCC |  |
| qF-NtFSD1d/e | NtFSD1d/e | GGCGGTGAGTTCTAGGCTTGA | 117 |
| qR-NtFSD1d/e |  | TCTCTAACTTCACCACCTGCTT |  |
| qF-NtFSD3 | NtFSD3a/b | GTTTGGACTTGTGGGAGCATGC | 178 |
| qR-NtFSD3 |  | GCATCCCAAGAGACAAGGTGATTC |  |
| qF-NtFSD1a | NtMSD1a | TTGGGCATTGATGTTTGGGAAC | 169 |
| qR-NtFSD1a |  | GTCCTCTGTATCGGTGTATCGC |  |
| qF-NtFSD1b/c | NtMSD1b/c | GTGGCTTGGTGTGGACAAAGA | 120 |
| qR-NtFSD1b/c |  | TGTTCCCAAACGTCTATTCCCA |  |
| F-NtCSD1a-*BamH* I | NtCSD1a | cgGGATCCATGGTGAAGGCCGTTGCCGT | 513 |
| R-NtCSD1a-*EcoR* I |  | cgGAATTCCTAGTAGCTGAGAATCTTAACGG |  |
| F-NtFSD1e-*BamH* I | NtFSD1e | cgGGATCCATGATGGCCGCTACAGCTTC | 924 |
| R-NtFSD1e-*EcoR* I |  | cgGAATTCTTATTCCGCATCAGAATCTG |  |
| F-NtMSD1b-*BamH* I | NtMSD1b | cgGGATCCATGGCACTACGAACCCTAGTG | 699 |
| R-NtMSD1b-*EcoR* I |  | cgGAATTCTCAAGGACATTCTTTCTCAT |  |
